# Radical Scavenging Activity and Physicochemical Properties of Aquafaba-Based Mayonnaises and Their Functional Ingredients

**DOI:** 10.3390/foods11081129

**Published:** 2022-04-14

**Authors:** Katarzyna Włodarczyk, Agnieszka Zienkiewicz, Aleksandra Szydłowska-Czerniak

**Affiliations:** 1 Department of Analytical Chemistry and Applied Spectroscopy, Faculty of Chemistry, Nicolaus Copernicus University in Toruń, Gagarina 7, 87-100 Toruń, Poland; kwlodarczyk@doktorant.umk.pl; 2 Centre for Modern Interdisciplinary Technologies, Nicolaus Copernicus University in Toruń, Wileńska 4, 87-100 Toruń, Poland; agazet@umk.pl

**Keywords:** aquafaba, cold-pressed oils, confocal laser scanning microscopy, egg replacement, physicochemical properties, radical scavenging activity, vegan mayonnaise

## Abstract

A plant-based diet has become more popular as a pathway to transition to more sustainable diets and personal health improvement in recent years. Hence, vegan mayonnaise can be proposed as an egg-free, allergy friendly vegan substitute for full-fat conventional mayonnaise. This study intends to evaluate the effect of aquafaba from chickpeas and blends of refined rapeseed oil (RRO) with different cold-pressed oils (10% of rapeseed oil—CPRO, sunflower oil—CPSO, linseed oil—CPLO or camelina oil—CPCO) on the radical scavenging, structural, emulsifying, and optical properties of novel vegan mayonnaise samples. Moreover, the functional properties and radical scavenging activity (RSA) of mayonnaise ingredients were evaluated. Aquafaba-based emulsions had a higher RSA than commercial vegan mayonnaise, determined by QUick, Easy, Novel, CHEap and Reproducible procedures using 2,2-diphenyl-1-picrylhydrazyl (QUENCHER-DPPH) and 2,2′-azino-bis(3-ethylbenzothiazoline-6-sulfonic acid) (QUENCHER-ABTS). Oxidative parameters such as peroxide values (PV), anisidine values (AnV), total oxidation (TOTOX) indexes and acid values (AV) of the proposed vegan mayonnaises were similar to those for commercial mayonnaises. Moreover, aquafaba-based samples had smaller oil droplet sizes than commercial vegan mayonnaise, which was observed using confocal laser scanning microscopy. The novel formulas developed in this study are promising alternatives to commercial vegan emulsions.

## 1. Introduction

Mayonnaise is semi-solid oil-in-water emulsion. Traditional mayonnaise is made from vegetable oil, egg yolk, vinegar, and spices during gentle mixing. Therefore, traditional mayonnaise as a high oil-containing product is susceptible to deterioration by fast, destructive oxidation of the unsaturated fats in its oil fraction. Oxidation processes reduce the nutritional value of fat-based products due to the loss of polyunsaturated lipids and vitamins that are beneficial to human health. However, the elimination of intrinsic (prooxidants content) and extrinsic factors (high temperature, light) and the addition of high-quality antioxidant ingredients can inhibit the oxidative reactions and increase the shelf life of mayonnaise [1]. On the other hand, mayonnaise ingredients, especially types of edible oils, fat replacers, and emulsifiers, strongly influence the physicochemical properties of the final products. Although the most common oils used to make mayonnaise are refined oils, such as rapeseed oil, sunflower oil, and soybean oil [2], the effect of various nut oils on the structure and physicochemical properties of gel-like emulsions was also investigated [3]. The gel-like emulsion with sunflower oil showed better stability than the gel-like emulsions of nuts oils due to the smaller particle size and higher viscosity. Moreover, mayonnaise made with linseed oil with a high level of linolenic acid (>50%) was more susceptible to lipid oxidation than mayonnaise with saturated medium-chain triglyceride oil [4]. However, Eidhin and O’Beirne [5] did not notice any changes in the oxidative stability of salad dressing and mayonnaises after the replacement of sunflower seed oil with refined camelina oil. In particular, many studies have reported that various cold-pressed oils (linseed, camelina, soybean, sunflower, rapeseed, corn, grapeseed, hemp, rice bran, pumpkin, walnut, rosehip, milk thistle, and black cumin) contain a higher level of natural antioxidants such as tocopherols (165.4–1036.0 mg/kg), phenols (5.1–1151.2 mg/kg), flavonoids (4.5–64.2 mg/kg), carotenoids (18.3–198.0 mg/kg), and squalene (0–1324.3 mg/kg) than refined vegetable oils [6,7,8,9,10]. Therefore, emulsions containing cold-pressed black cumin oil and cold-pressed rice bran oil had a better oxidative status (lower amounts of primary and secondary oxidation products) and antioxidant properties (higher total phenolics content and antioxidant potential determined by 2,2-diphenyl-1-picrylhydrazyl (DPPH) assay) than control emulsions without cold-pressed oils [10,11].

Consequently, lipid oxidation in emulsions depends also on the type of emulsifier. It is well known that egg yolk is the most popular emulsifier used in traditional mayonnaise formulation. However, eggs consumption increases the risk of cardiovascular disease, particularly among diabetic patients [12,13,14], while today’s consumers express greater concern about the quality and health benefits of eaten products. Lately, consumption of this product has decreased to promote environmental care and animal welfare. The mean daily water consumption footprint per capita in the European Union countries for eggs and egg products was approximately 1.5 times higher than this result for legumes, nuts, oilseeds and spices [15]. One other reason to reduce the consumption of eggs is an allergy caused by an allergic reaction to their proteins. Unfortunately, raw eggs present in mayonnaise are more likely to cause an allergic reaction than small amounts of cooked eggs. Egg allergy is one of the most common food allergies in infancy and small children, with a prevalence of up to 3.2% in the USA and Western Europe [16]. However, an allergy to eggs is rarely diagnosed in adults. Therefore, egg yolk in mayonnaise samples has often been replaced with plant protein isolates [17], canola protein [18], milk protein [19], soy milk [20], peanut and sesame meal milk [21], and Arabic gum [22]. Moreover, the effect of wastewater after cooking chickpeas (aquafaba) on the texture and physicochemical properties of plant-based mayonnaise was investigated [23]. Recently, the application of one of the most popular pulses cooking water (PCW)—aquafaba as a food ingredient has successfully broadened the list of new egg replacers [24]. Damian et al. [25] reported that PCW was a stable emulsifier and a rich source of phenolic compounds (0.3–0.7 mg/mL) and saponins (8–12 mg/mL). The amount of leached proteins and saponins impacted the foaming and gelling abilities of PCW. Other applications of aquafaba are in bakery products, dressing, dip, ice cream, legume-based cheese, and legume-based dairy substitutes [26]. Interestingly, chickpea ranks third among total legume production worldwide, after beans and peas and accounting for 10.1 million tons annually [27].

In recent years, the functional properties of aquafaba such as its emulsifying capacity, foaming ability, gelling attributed to its composition of proteins, carbohydrates, polysaccharide-protein complexes, coacervates, saponins, and phenolic compounds have been used in various formulations for vegans [28,29,30].

On the other hand, antioxidant compounds added to fat-based foods counteract lipid oxidation by acting as reducing agents, free radical scavengers and inactivators of prooxidants. Therefore, the antioxidant potential of emulsions enriched with new functional ingredients with antioxidant properties determines their quality and storage stability.

In this study, understanding the relationship between emulsion properties (mainly oxidative stability) and radical scavenging activity (RSA) is essential to improving the quality of mayonnaise production. To the best of our knowledge, there was no reference to the determination of the antioxidant properties of eggless mayonnaise containing aquafaba as a functional replacer. Therefore, the present work focused on the evaluation of radical scavenging characteristics, oxidation stability, microstructures, and optical properties of vegan mayonnaises containing aquafaba from chickpeas and blends of refined rapeseed oil (RRO) with different cold-pressed oils as key ingredients. The radical scavenging and physicochemical properties of the prepared emulsions were characterized and compared with commercial samples (egg yolk-based and plant-based mayonnaises). The modified QUick, Easy, New, CHEap, and Reproducible (QUENCHER) procedure was applied for an evaluation of the RSA of mayonnaises, whereas the radical scavenging characteristics of ingredients were analyzed by two conventional 2,2-diphenyl-1-picrylhydrazyl (DPPH) and 2,2′-azino-bis(3-ethylbenzothiazoline-6-sulfonic acid) (ABTS) methods. Moreover, the emulsifying properties of aquafaba and fatty acid composition (FAC) of cold-pressed oils were estimated and discussed. Finally, a principal component analysis (PCA) was applied to check the differences and similarities among all of the studied mayonnaise samples.

## 2. Materials and Methods

### 2.1. Reagents and Materials

Chickpeas (*Cicer arietinum* L.), mustard, salt, vinegar, sugar, nutritional yeast, and eggs were purchased in a local market. Refined rapeseed oil (RRO), cold-pressed rapeseed oil from *Brassica napus* L. (CPRO), cold-pressed sunflower oil from *Helianthus* L. (CPSO), cold-pressed linseed oil from *Linum usitatissimum* L. (CPLO) and cold-pressed camelina oil from *Camelina sativa* L. (CPCO) were kindly provided by a local vegetable oil factory in the original packing (1 L of RRO in polyethylene terephthalate (PET) bottle, 250 mL of cold-pressed oils in amber-colored glass bottles, marasca type).

Additionally, three mayonnaises (MT1, MT2—traditional recipe mayonnaises (full-fat, 76 and 68%, respectively, and MV—vegan mayonnaise containing 35% fat) packed in colorless glass jars were supplied directly after production by two different manufacturers representing the top-selling brands in the Polish market.

All oil and mayonnaise samples were within their stated shelf lives and stored in a refrigerator at 4 °C until analysis (no longer than 4 days after opening the original packaging).

All chemicals of analytical grade were purchased from Sigma-Aldrich (Poznań, Poland). Redistilled water was used for the preparation of solutions.

### 2.2. Preparation of Mayonnaise Samples

**Figure 1:**
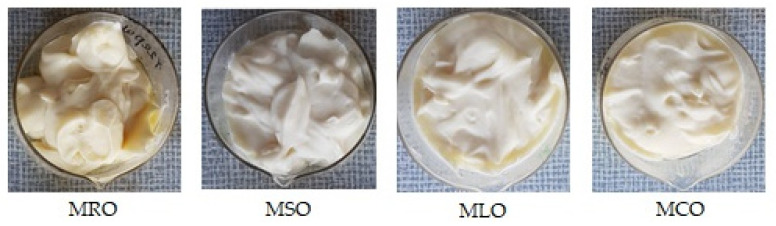
The appearance of the prepared aquafaba-based mayonnaise samples with blends of refined rapeseed oil and cold-pressed rapeseed oil (MRO), cold-pressed sunflower oil (MSO), cold-pressed linseed oil (MLO), and cold-pressed camelina oil (MCO).

Aquafaba, the liquid from the chickpea jars, was separated using a stainless-steel mesh kitchen strainer. A representative sample of aquafaba was taken for analysis and mayonnaise preparation. The content of each ingredient for the preparation of mayonnaise samples was selected based on preliminary tests. Four mayonnaise batches (MRO, MSO, MLO and MCO) of 200 g were prepared according to the procedure described by Raikos et al. [23] with some modifications using the ingredients listed in Table 1. Aquafaba was mixed with mustard, vinegar, nutritional yeast, salt, and sugar using a high mixing bowl and a stick blender (BOSH^®^ MSM67160, Robert Bosch GmbH, Gerlingen, Germany). Then, blends of RRO with each cold-pressed oil were gradually and slowly added to the aqueous mixture during the blending procedure to achieve the proper consistency. Emulsions were homogenized for 5 min utilizing a homogenizer (DT basic, Yellow Line, IKA^®^-Werke GmbH & Co., KG, Staufen, Germany) at 8000 rpm. The appearance of the prepared aquafaba-based emulsions is shown in Figure 1. Mayonnaises were packaged into colorless glass jars and stored at 4 °C in a refrigerator until analyses (no longer than 4 days).

### 2.3. Characterization of Mayonnaise Ingredients

#### 2.3.1. Determination of Emulsifying Properties

The emulsifying activity index (EAI) and the emulsion stability index (ESI) were determined according to the procedure described by Cheung et al. [31] but developed initially by Pearce and Kinsella [32]. In brief, 5.0 g of aquafaba or egg yolk was homogenized with 5.0 g of RRO using a homogenizer at a speed of 8000 rpm for 5 min. Then, a 50 µL aliquot of the emulsion was diluted to 7.5 mL of 0.1% sodium dodecyl sulphate (SDS) and vortexed using a classic vortex mixer (Velp Scientifica Srl, Usmate (MB), Italy) for 10 s. The absorbance of the diluted emulsion samples was measured at λ = 500 nm by a Hitachi U-2900 spectrophotometer (Tokyo, Japan). The EAI and ESI were calculated using the following equations:

(1) $$\mathrm{EAI}\left(\frac{m^{2}}{g}\right)=\frac{2\;\cdot \;2.303\;\cdot \;A_{0}\;\cdot \;N}{c\;\cdot \;\varphi \;\cdot \;\phi \;\cdot \;10000}$$

(2) $$\mathrm{ESI}\;\left(\min\right)=\frac{A_{0}}{A_{0}-A_{10}}\;\cdot \;t$$

where, *A*_0_ and *A*_10_ are the absorbance values measured at an initial time, and after 10 min, respectively, *t* is the time interval (10 min), *N* is the dilution factor, *c* is the protein concentration (g/mL), *φ* is the oil volume fraction of the emulsion and *ϕ* is an optical path (1 cm).

#### 2.3.2. Determination of Protein Concentration

The protein concentration was determined with the Kjeldahl method, according to the official Polish Standard Method PN-75/A-04018 [33]. The protein concentration was estimated as the nitrogen content multiplied by a conversion factor of 6.25.

#### 2.3.3. Determination of Fatty Acid Composition

Fatty acid profiles for RRO and all cold-pressed vegetable oils (CPRO, CPSO, CPLO and CPCO) were determined in accordance with the official method ISO 5508 [34]. Fatty acid methyl esters (FAMEs) were prepared by the transesterification of oil samples carried out in methanol using potassium hydroxide as a base and the derivatization of fatty acids was conducted following the procedure described by ISO 5509 [35].

The quantification of fatty acids was performed by applying a gas chromatograph (HP 5890 GC) equipped with a flame-ionization detector (FID) (Hewlett-Packard, Avondale, PA, USA) and a high polar capillary column BPX 70 (60 m × 0.25 mm, 0.25 μm). The temperatures of the injector and detector were adjusted to 250 °C, while the oven temperature program was as follows: heating from 150 to 210 °C at 1.3 °C/min and holding at 210 °C for 5 min. The carrier gas was helium at a flow rate of 0.6 mL/min.

The identification of fatty acids was accomplished using external FAME standards, and the results are presented as weight percentages of total fatty acids.

#### 2.3.4. Determination of Radical Scavenging Activity

The RSA values of aquafaba, oils (RRO, CPRO, CPSO, CPLO and CPCO), mustard and nutritional yeast were analyzed spectrophotometrically by DPPH and ABTS methods according to the modified procedures previously described in detail [36].

The liquid aquafaba sample was dissolved in methanol and used directly for RSA measurements.

The test tubes with oils (3.00 g), mustard (2.00 g) and nutritional yeast (0.50 g) with methanol (5 mL) were shaken for 30 min using a shaker SK-0 330-PRO (CHEMLAND, Stargard Szczeciński, Poland) at room temperature. The extracts were separated from oils in a freezer (−20 °C, 30 min) and transferred quantitatively into glass bottles. Each studied sample was extracted in triplicate, and extracts were stored in a refrigerator prior to RSA analysis.

In the case of the DPPH test, 0.3 mL of 0.1% methanolic solution of aquafaba (*v*/*v*) or 0.2–0.5 mL of the methanolic extracts of oils, mustard and nutritional yeast were added to 1.8–1.5 methanol and 0.5 mL of DPPH methanolic solution (304 μmol/L). The mixtures were shaken vigorously and left in darkness for 15 min. The absorbance of each sample was measured at 517 nm against a reagent blank (2 mL of methanol and 0.5 mL of DPPH methanolic solution) using a Hitachi U-2900 spectrophotometer in a 1 cm glass cell.

For the ABTS assay, 0.1 mL of 0.1% methanolic solution of aquafaba (*v*/*v*) or 0.1–0.3 mL of the methanolic extracts of oils, mustard and nutritional yeast were added to 2.4–2.2 mL of ABTS^•+^ reagent (diluted with ethanol to an absorbance of 0.70 ± 0.02 at 734 nm). The obtained mixtures were incubated at 30 °C for 1 min and the absorbance of each sample was measured at 734 nm against a reagent blank (2.5 mL of ABTS^•+^ solution).

The RSA values were expressed as micromoles of Trolox equivalents (TE) per 100 g of the studied sample.

### 2.4. Characterization of Mayonnaise Samples

#### 2.4.1. Determination of Radical Scavenging Activity

The QUENCHER-DPPH and QUENCHER-ABTS extraction-free procedures were applied for a direct evaluation of the RSA of the proposed vegan and commercial mayonnaises.

For the QUENCHER-DPPH assay, 0.0300–0.0500 g of the mayonnaise samples was transferred into centrifuge tubes. The reaction was started by adding 6 mL of DPPH solution (60.8 µmol/L). The mixture was vortexed for 5 min and left in darkness for 15 min. The samples were centrifuged at 3120× g (centrifuge MPW-54, MPW MED. INSTRUMENTS, Warsaw, Poland) for 3 min and the absorbance of optically clear supernatant was measured at 517 nm.

For the QUENCHER-ABTS assay, 0.0200–0.0300 g of the mayonnaise samples was weighed to centrifuge tubes and the reaction was started by adding 6 mL of ABTS^•+^ reagent (diluted with ethanol to an absorbance of 0.70 ± 0.02 at 734 nm). The mixtures were vortexed for 5 min and centrifuged at 3120× *g* for 3 min. The absorbance of clear supernatants was measured at 734 nm against a reagent blank (2.5 mL of ABTS^•+^ solution).

#### 2.4.2. Determination of Oxidative Stability

The lipid phase of each mayonnaise sample was separated according to the procedure [37] for an analysis of oxidative stability. Mayonnaises were frozen at −18 °C for 12 h and then thawed for 12 h at 4 °C to break the emulsion. The mixture was centrifuged for 5 min. Each lipid phase, separated from the emulsion residue, was stored in a closed glass flask in the refrigerator prior to further analysis.

The peroxide value (PV), anisidine value (AnV) and acid value (AV) of the lipid phases were determined to estimate the formation of primary and secondary oxidation products as well as free fatty acids, respectively, that affect the rancidity and the mayonnaise stability.

The PV was measured by iodometric titration according to the official procedure ISO 3960:2017 [38] and was expressed as milliequivalents of active oxygen per kilogram of lipid phase (mEq O_2_/kg).

The AnV was analyzed according to the ISO 6885:2016 method [39].

The oxidation state of the lipid phase given by the TOTOX index was calculated according to the formula: (TOTOX = 2PV + AnV).

However, the AV was analyzed according to the ISO 660 (1996) method [40].

#### 2.4.3. Microstructure

Commercial mayonnaises (MT1, MT2, MV) and freshly made emulsions (MRO, MSO, MLO, MCO) were observed under an Olympus FluoView 3000 confocal laser scanning microscope (Olympus, Tokyo, Japan). All samples were prepared by adding 0.01% Nile red and analyzed using a diode 488 nm laser (excitation). The area of oil droplets (ODs) from each sample was quantified from 10 to 15 confocal images acquired by FluoView software (Olympus), corresponding to ∼1000 ODs by using ImageJ software (www.imagej.nih.gov) (accessed on 6 November 2020). Data point-box plots were generated with Origin Pro software (OriginLab Corporation, Northampton, MA, USA).

#### 2.4.4. Color Parameters

Before an analysis of the color parameters, all mayonnaise samples were thoroughly mixed. The color was measured using a MICRO-COLOR II LCM 6 spectrophotometer (Dr. Bruno Lange GmbH & Co. KG, Berlin, Germany) based on three color coordinates, namely L*, a*, b*. The color values were expressed as L* (whiteness or brightness/darkness), a* (redness/greenness) and b* (yellowness/blueness).

### 2.5. Statistical Analysis

The measurements of the oxidative parameters of lipid phase, emulsifying properties, and protein content in emulsifiers were performed in triplicate on the same day to verify the repeatability of the obtained results. The RSA for ingredients and emulsions, FAC of vegetable oils, and color parameters of mayonnaises were measured in five replications. All results were presented as mean (c) ± standard deviation (SD). A one-way analysis of variance (ANOVA), which was followed by the Tukey’s post hoc test, was performed to analyze the significant differences between data (*p* < 0.05). Moreover, PCA was employed to study the clustering and differentiation of seven mayonnaise samples based on QUENCHER-DPPH, QUENCHER-ABTS, PV, AnV, TOTOX, AV, area of ODs, L*, a* and b* results. The scores and loadings of the data analyzed by PCA were displayed as a bi-plot. Statistical analyses of data were carried out using the Statistica 8.0 software (StatSoft, Tulsa, OK, USA).

## 3. Results and Discussion

### 3.1. Characterization of Mayonnaise Ingredients

#### 3.1.1. Emulsifying Properties and Protein Content

Two indexes, namely the emulsifying activity (EAI) and emulsifying stability (ESI) were used to characterize the emulsifying properties of aquafaba. The obtained EAI and ESI results and protein content in aquafaba were compared with those values for an egg yolk (Table 2).

The EAI is an oil/water interface area stabilized per unit weight of protein. As can be seen, the EAI of aquafaba (13.75 m^2^/g) is almost 8-fold higher than the EAI of egg yolk (1.78 m^2^/g). This calculated EAI result for egg yolk was significantly lower than previously published values ranging between 24.5 and 30.5 m^2^/g [41,42]. Moreover, Meurer et al. [29] observed a high stability of egg yolk-based emulsion during 4 days (EAI = 100%). In general, differences between the EAI results can be caused by various types and concentrations of proteins, their hydrophobicity, unfolding ability, the treatment and storage conditions of egg yolks, as well as procedures and equipment used in emulsions production. It is probable that the studied egg yolk contained a lower amount of proteins with lower solubility and structural unfolding than the egg yolk proteins investigated by other authors [41,42]. A higher EAI of aquafaba proteins could be attributed to a combination of the less compact structure and higher solubility, which enhanced the ability to form interfacial membranes around the oil droplets.

However, the ESI represents a decrease in turbidity of a diluted emulsion over time. This parameter depends on the resistance of proteins to coalescence, sedimentation, flocculation and creaming over a certain period [43]. It is noteworthy that the ESI of egg yolk-based emulsion was over 110-fold higher than the ESI of vegetable emulsion produced by aquafaba (Table 2). The EAI and ESI results obtained for aquafaba (EAI = 13.75 m^2^/g and ESI = 20.92 min) were comparable with those reported by other authors (EAI = 12–38.6 m^2^/g and ESI = 15–25 min) [25,28]. The emulsifying properties of aquafaba were highly dependent on chickpea cultivar, canning process conditions and additives [24,44]. Additionally, amounts of proteins, carbohydrates, saponins, and phenolic compounds, as well as thermal processes, affected the functional properties of aquafaba [45]. It is known that during emulsion generation, proteins reduced the interfacial tension at the oil–water interface due to the presence of hydrophobic and hydrophilic groups. Although the studied aquafaba had a significantly lower protein content (1.26%) than egg yolk (16.12%), aquafaba proteins aggregated at the water–oil interface and formed an intermolecular cohesive film with enough elasticity to stabilize emulsions.

At the same time, polysaccharides can stabilize the emulsion and prevent flocculation and coalescence [46]. Furthermore, proteins and polysaccharides present in aquafaba can be bound by bioactive substances such as saponins and phenolic compounds, causing changes in the emulsion capacity of this natural emulsifier [25]. Therefore, a low ESI value for aquafaba can be related to its high RSA determined by DPPH and ABTS methods and discussed in Section 3.1.3.

For comparison, Günal-Köroğlu et al. [47] observed that the EAI (13.9–41.6 m^2^/g) and ESI (26.1–98.6 min) for emulsions of lentil protein isolate–phenolic solutions were inversely proportional to the concentrations of gallic acid (0.05–0.25 mg/mL) and phenolic extracts from yellow and red onion skin (0.10–0.50 mg/mL). On the other hand, the amphiphilic structure of saponins generated smaller oil droplets (ODs) during homogenization by lowering interfacial tension [48].

#### 3.1.2. Fatty Acid Compositions of Vegetable Oils

The fatty acid compositions of oils used to prepare new vegan mayonnaise samples are presented in Table 3.

Tukey’s post hoc test indicated significant differences in the fatty acid percentages of the studied cold-pressed vegetable oils and RRO. Each investigated vegetable oil has a specific fatty acid profile depending on the plant sources. It is noteworthy that all vegetable oils contained a low amount of saturated fatty acids (SAFA = 6.9–10.6%) and a high level of monounsaturated fatty acids (MUFA = 15.7–64.6%) and polyunsaturated fatty acids (PUFA = 27.4–73.8%).

The major SAFA were palmitic acid (C16:0) and stearic acid (C18:0) which were determined in the highest concentrations in CPSO (6.6%) and CPCO (4.1%) samples, respectively. Rapeseed oils (RRO and CPRO) were the richest source of oleic acid (18:1n-9 = 62.2–63.4%), followed by linoleic acid (18:2n-6 = 19.3–19.4%).

On the contrary, linoleic acid was present at the highest level (58.9%) in CPSO, while a moderate content of oleic acid (29%) was found in this oil (Table 3). However, linolenic acid (C18:3n-3 = 30.5–57.5%) was the principal PUFA for two of the cold-pressed oils, CPLO and CPCO. Nevertheless, CPCO had an approximately two times higher C18:3 percentage than CPLO.

As can be seen, CPSO, CPLO and CPCO revealed the highest PUFA content (49.7–73.8%), whereas MUFA accounted for more than 60% of total fatty acids in CPRO and RRO.

It is known that the consumption of vegetable oils with high amounts of unsaturated fatty acids exert a substantial impact on human health, mainly in the prevention of cardiovascular and cancer diseases. On the other hand, vegetable oils rich in PUFA are sensitive to oxidative damage during storage and processing [36].

Our previous studies reported similar results of FAC for rapeseed oils (SAFA = 6.91–7.58%, MUFA = 64.14–66.14%, PUFA = 27.22–30.17%) [36]. However, Symoniuk et al. [6] obtained a higher level of PUFA (64.30–77.42%) in linseed oils.

For comparison, Ratusz et al. [7] studied the quality characteristics of commercial cold-pressed camelina oils and reported somewhat different concentrations of 7.4–10.1% for SAFA, 31.3–36.3% for MUFA, and 55.2–58.4% for PUFA. Specifically, the fatty acid profiles depend on both genetic and environmental factors.

#### 3.1.3. Radical Scavenging Activity of Mayonnaise Ingredients

The radical scavenging properties of mayonnaise ingredients were analyzed by the DPPH and ABTS assays, and the obtained RSA results are presented in Table 4.

Among the investigated ingredients, nutritional yeast and mustard had the highest DPPH (1687 and 1129 μmol TE/100 g) and ABTS (9192 and 4985 μmol TE/100 g) values. For comparison, Bors et al. [49] reported that the RSA of mustard paste determined using the DPPH method ranged between 23.4–23.7%.

The radical scavenging properties of aquafaba (DPPH = 437 μmol TE/100 g and ABTS = 2097 μmol TE/100 g) were higher than reported by previously published data (0.15–0.38 μmol TE/g) [50]. This variability in total antioxidant content in chickpeas and aquafaba can be explained by the prolonged time of contact of chickpeas with aquafaba in the jar as well as the influence of other factors such as genetic, agronomic, environmental and technological factors. It is known that the ABTS assay is applicable to both hydrophilic and lipophilic antioxidant systems, whereas the DPPH assay is more suited to hydrophobic systems. It can be noted that the ABTS value of aquafaba (2097 μmol TE/100 g) was five times higher than the DPPH value (437 μmol TE/100 g), probably due to water-soluble antioxidants being predominant in the aquafaba containing 95% water (Table 4).

Moreover, the ABTS results (588–738 μmol TE/100 g) of oils used to make vegan mayonnaise samples were higher than DPPH values (272–391 μmol TE/100 g). Insignificant differences for ABTS values of all the studied cold-pressed vegetable oils were observed, whereas RRO, CPRO and CPCO had significantly higher DPPH results than CPSO and CPLO (Tukey’s post hoc test, Table 4). Interestingly, rapeseed and camelina cold-pressed oils were found to be richer sources of antioxidants than cold-pressed sunflower and linseed oils. A similar decrease in DPPH values for cold-pressed oils (rapeseed > sunflower > flax and camelina > flaxseed) was reported by Siger et al. [8] and Grajzer et al. [9].

### 3.2. Characterization of Mayonnaise Samples

#### 3.2.1. Radical Scavenging Activity

The QUENCHER-DPPH and QUENCHER-ABTS methods were proposed for the first time to directly measure the RSA of the developed vegan egg-free mayonnaises containing blends of RRO with different cold-pressed oils and to compare their radical scavenging properties with that of commercial mayonnaises.

These analytical methodologies rely on the surface reaction phenomenon between mayonnaise samples with bound and free radical scavengers with organic DPPH radical and ABTS cation radical solutions. The QUENCHER method, as a cost-saving and extraction-free procedure, is highly relevant and recommended for the fat industrial laboratory to quickly control the antioxidant properties of emulsions.

**Figure 2:**
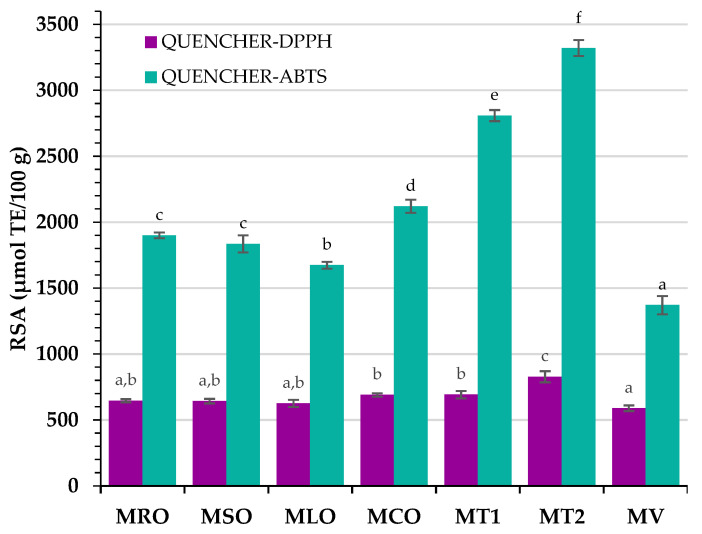
Radical scavenging activity of aquafaba-based mayonnaises with blends of refined rapeseed oil and cold-pressed rapeseed oil (MRO), cold-pressed sunflower oil (MSO), cold-pressed linseed oil (MLO), and cold-pressed camelina oil (MCO); MT1—commercial egg yolk mayonnaise from producer 1; MT2—commercial egg yolk mayonnaise from producer 2; MV—commercial vegan mayonnaise; QUENCHER-DPPH—QUick, Easy, Novel, CHEap and Reproducible-2,2-diphenyl- 1-picrylhydrazyl method; QUENCHER-ABTS—QUick, Easy, Novel, CHEap and Reproducible-2,2′-azino-bis(3-ethylbenzothiazoline-6-sulfonic acid) method. Bars with different letters (a–f) indicate significant differences between radical scavenging activity of mayonnaises (Tukey’s post hoc test, *p* < 0.05).

The QUENCHER-DPPH and QUENCHER-ABTS results for all of the studied mayonnaise samples are presented in Figure 2.

It is noteworthy that the richest sources of hydrophilic and lipophilic antioxidants were traditional full fat mayonnaises (QUENCHER-ABTS = 3320 μmol TE/100 g and 2808 μmol TE/100 g for MT2 and MT1, respectively), while MT2 had a higher content of lipophilic antioxidants, as determined by the QUENCHER-DPPH test (828 μmol TE/100 g), than MT1 (DPPH = 692 μmol TE/100 g). In contrast, the store-bought plant-based mayonnaise (MV) revealed the lowest QUENCHER-DPPH (589 μmol TE/100 g) and QUENCHER-ABTS (1371 μmol TE/100 g) values (Figure 2). However, among aquafaba-based mayonnaises, samples containing blends of RRO with cold-pressed oils had similar DPPH results, whereas only the ABTS values for MRO and MSO did not differ significantly (Tukey’s post hoc test, Figure 2). The lowest effectiveness of MLO to radical scavenging can be explained by the lowest DPPH (270 μmol TE/100 g) for CPLO among all cold-pressed oils (Table 4). It is worth noting that the addition of CPCO into the MCO sample caused a significant increase in QUENCHER-DPPH and QUENCHER-ABTS results. Moreover, nutritional yeast and mustard had the highest radical scavenging properties (Table 4) which probably increased the RSA of aquafaba-based mayonnaise compared to commercially available MV mayonnaise.

For comparison, the methanolic extract of traditional mayonnaise had an RSA (DPPH = 267 μmol TE/100 g and ABTS = 856 μmol TE/100 g [51]) that was approximately three-fold lower than commercial samples measured by the QUENCHER procedure proposed in this study. Moreover, DPPH (78–134 μmol TE/100 g) and ABTS results (463–613 μmol TE/100 g) for methanol:water (70:30) extracts of vegan mayonnaises based on soy milk [52] were lower than our QUENCHER-DPPH values (589–828 μmol TE/100 g) and QUENCHER-ABTS values (1371–3320 μmol TE/100 g). The higher radical scavenging properties of mayonnaises determined by the QUENCHER procedure can be explained as the proposed analytical method detects both the extractable and non-extractable compounds present in the analyzed samples.

#### 3.2.2. Oxidative Status

The oxidative status of the oil phase of each mayonnaise sample was evaluated by characteristic values such as PV, AnV, TOTOX and AV (Table 5).

The PV shows the degree of peroxidation and measures the amount of total peroxides, whereas the content of aldehyde carbonyl bonds formed during secondary lipid oxidation is evaluated as AnV. PV and AnV measurements are commonly used together to determine the total extent of oxidation by the TOTOX index. Moreover, AV is an important variable for the quality of the fat-based product because it reveals free fatty acid content affecting its oxidative ageing.

As can be seen, PV results were significantly different in different mayonnaise samples (Tukey’s post hoc test, Table 5). The lowest amount of primary oxidation products was in MT2 (PV = 0.82 mEq O_2_/kg), while vegan mayonnaise (MV) had the highest PV = 4.61 mEq O_2_/kg. This can be explained by the highest amount of total antioxidants in the MT2 sample (QUENCHER-DPPH = 828 μmol TE/100 g and QUENCHER-ABTS = 3320 μmol TE/100 g, Figure 2), which most effectively inhibited harmful oxidation reactions. However, the highest PV for MV can be attributed to the lowest antioxidant potential (QUENCHER-DPPH = 589 μmol TE/100 g and QUENCHER-ABTS = 1371 μmol TE/100 g), preventing the peroxidation in polyunsaturated fatty acids of the oil phase. Moreover, the addition of CPLO with a high level of unsaturated fatty acids (MUFA = 33.8% and PUFA = 49.7%, Table 3) sensitive to peroxidation raised the susceptibility of mayonnaise to oxidation. Thus, MLO had a high level of PV (2.27 mEq O_2_/kg) and AnV (2.95) in its fat phase (Table 5). Meanwhile, the presence of resistant SAFA (7.0–10.6%) to degradation processes and a high percentage of MUFA (29.1–64.6%) in CPRO, and CPSO improved the oxidative stability of MRO and MSO samples (PV and AnV ranged between 1.43–1.90 mEq O_2_/kg and 1.12–1.39, respectively).

Unexpectedly, low amounts of primary (PV = 1.19 mEq O_2_/kg) and secondary (AnV = 1.55) oxidative products in the oil phase of MCO with the highest content of PUFA (73.8%, Table 3) can be explained by the high radical scavenging properties of CPCO (DPPH = 387 μmol TE/100 g and ABTS = 706 μmol TE/100 g, Table 4) and MCO (QUENCHER-DPPH = 690 μmol TE/100 g and QUENCHER-ABTS = 2121 μmol TE/100 g, Figure 2). The antioxidant compounds in cold-pressed oils may act as antioxidants and prevent or delay the lipid oxidation of mayonnaises.

For comparison, the effect of cold-pressed black cumin oil (CPBCO) on the oxidative stability of traditional mayonnaise was evaluated. At the end of storage for 4 weeks at 20 °C, mayonnaise with 20% of CPBCO had a lower content of primary oxidation products (PV = 17.66 mEq O_2_/kg) than the control sample (36.07 mEq O_2_/kg) [10]. Moreover, substituting egg yolk with milk protein and adding high levels of fish oil to the mayonnaise samples raised the PV results to approximately 75 and 25 mEq O_2_/kg at 20 °C and 2 °C of storage, respectively [19].

It is noteworthy that the best stability revealed the MT2 sample with the lowest TOTOX index (2.40), which combines the amount of primary with secondary oxidation products. In contrast, the MV sample containing the lowest total antioxidants level (QUENCHER-DPPH = 589 μmol TE/ 100 g and QUENCHER-ABTS = 1371 μmol TE/100 g, Figure 2) was characterized with the highest TOTOX value = 12.29 (Table 5).

Additionally, aquafaba-based mayonnaises had a significantly lower AV (0.11–0.26 mg KOH/g) than commercial mayonnaise samples (AV = 0.49–0.52 mg KOH/g).

Similarly, commercial mayonnaise revealed higher AV (5.39 mg KOH/g) than samples made with lemon juice and a mix of lemon juice and vinegar (AV = 1.68–3.37 mg KOH/g), which can be explained by non-specific reactions during AV measurements [53].

#### 3.2.3. Microstructure

Previous studies clearly demonstrated that the size of oil droplets (ODs) is one of the most essential parameters influencing mayonnaise stability, texture, and taste [54]. Mayonnaises characterized by small ODs usually possess significantly increased viscosity and stability. The size of ODs depends on the homogenization approach, including its duration and intensity, mayonnaise composition, and ingredient viscosity [55].

**Figure 3:**
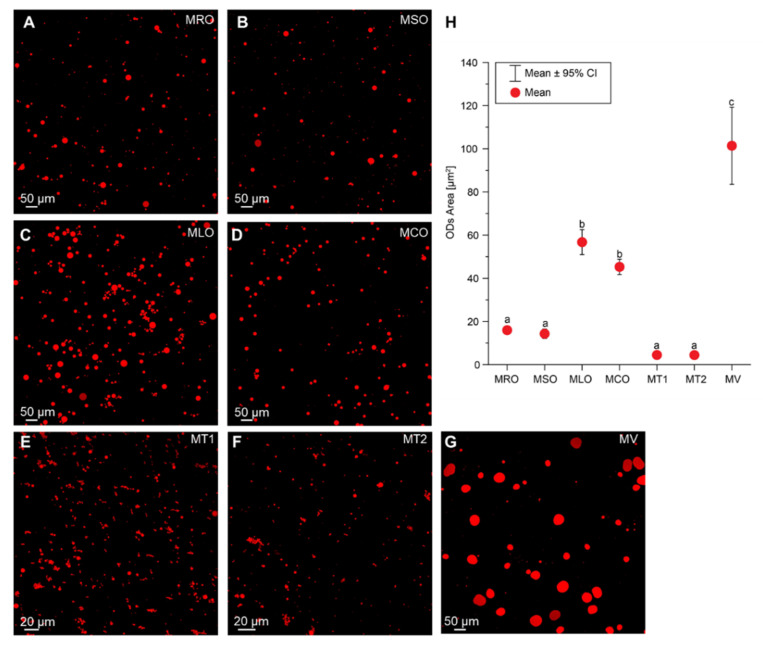
(**A**–**G**) Representative images of oil droplets (ODs) stained with Nile red. (**H**) The data point-box plot showing a mean area of ODs quantified with imaging software (ImageJ). A statistical analysis was performed by a one-way analysis of variance (ANOVA) with Tukey’s post hoc test (*p* < 0.05). Different letters (a–c) indicate significant differences with *p* < 0.05 between aquafaba-based mayonnaises with blends of refined rapeseed oil and cold-pressed rapeseed oil (MRO), cold-pressed sunflower oil (MSO), cold-pressed linseed oil (MLO), and cold-pressed camelina oil (MCO); MT1—commercial egg yolk mayonnaise from producer 1; MT2—commercial egg yolk mayonnaise from producer 2; MV—commercial vegan mayonnaise.

In our studies, the CLMS images revealed differences in the size of ODs between analyzed emulsions (Figure 3A–G). The largest ODs were observed for MV and smallest ODs were found for MT1 and MT2. Recently, it was reported that aquafaba-emulsifying properties caused the presence of larger ODs compared to the egg-yolk-based mayonnaise [23,30]. The size of ODs likely depends on the size of emulsifier molecules which in the egg yolk are much smaller compared to aquafaba [30]. He et al. [30] demonstrated that the average of ODs size (*d*_43_) of mayonnaise analogue prepared using freeze-dried or spray-dried aquafaba was significantly larger, at 222 ± 9 μm^2^ and 97.3 ± 16 μm^2^, respectively, compared to egg yolk based mayonnaise (9.02 ± 1 μm^2^). Our results seem to confirm that the addition of plant-based proteins, such as pea protein, results in larger average areas of ODs (101.40 µm^2^) in cases of MV when compared to egg yolk-based mayonnaise MT1 and MT2, where the average areas of ODs were 4.47 µm^2^ and 4.44 µm^2^, respectively. We also cannot exclude that the larger size of ODs in MV might be the result of low oil content (35%) in comparison to MT1 and MT2 (around 70%).

The use of equal amounts of aquafaba—23.70% and different oil composition was accompanied by the presence of significantly larger ODs in MCO (45.28 µm^2^) and in MLO (56.75 µm^2^). In turn, ODs in MRO and in MSO showed an average area of 15.96 µm^2^ and 14.29 µm^2^, respectively (Figure 3H). The observed variations in ODs size between the analyzed mayonnaises could reflect the differences in their lipidic composition.

#### 3.2.4. Color

It is crucial to control the color of mayonnaise samples to obtain the desired color that most affects the consumer’s willingness to purchase or taste a new product. Therefore, values of lightness (L*), redness (a*) and yellowness (b*) of the prepared vegan mayonnaise samples are listed in Table 6.

The Tukey’s post hoc test indicated that the color parameters for mayonnaises with blends of RRO and various cold-pressed oils significantly differ from each other. The highest lightness value (L* = 48.7–47.7) had MRO, MT1 and MT2, respectively. It can be caused by the smaller OD sizes in these samples (Figure 3). Due to light scattering, mayonnaises with smaller OD sizes are whiter, and also the intensity of the yellow color is lower [56]. It is noteworthy that store-bought samples had higher redness values (a* = 2.2–3.4) than the prepared aquafaba-based samples (a* = −0.3–0.9). High yellowness values (b* = 9.0–11.2) for commercial samples MT1 and MT2 containing a high egg yolk content can be preferable by consumers. The highest b* value (11.3) for the MV sample was probably caused by the addition of carotenoids. High b* values (6.7–10.9) and low a* values (−0.3–0.9) for MRO, MSO, MLO and MCO samples with cold-pressed oils can be explained by the presence of natural pigments, mainly carotenoids and chlorophyll, in added press-cold oils.

For comparison, the a* and b* values of mayonnaises with different oil-to-aquafaba (O/A) ratios increased significantly for higher oil contents in mayonnaises (a* = 3.5–4.1 and b* = 36.5–39.9 for O/A = 80/15, while a* = 2.6–2.9 and b* = 30.5–31.8 for O/A = 70/25). Moreover, mayonnaise samples with smaller OD sizes had greater brightness values [23].

### 3.3. Principal Component Analysis

PCA was used to observe any possible groups within the commercial and prepared mayonnaises as well as to reveal the interrelationships among variables (QUENCHER-DPPH, QUENCHER-ABTS, PV, AnV, TOTOX, AV, area of ODs, L*, a* and b*) that mainly influence the similarities and differences of the analyzed samples. The first two principal components took into account 85.86% (PC1 = 57.80% and PC2 = 28.06%, respectively) of the total variation.

**Figure 4:**
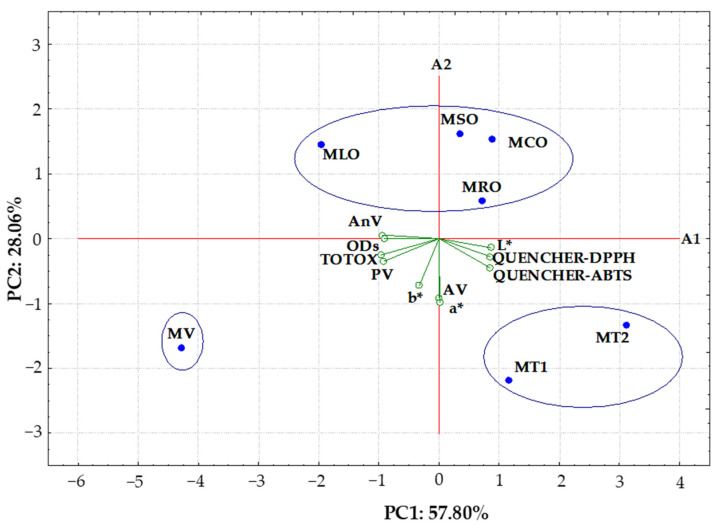
Biplot of scores and loadings of data obtained from radical scavenging properties (QUENCHER-DPPH, QUENCHER-ABTS), oxidation stability (PV—peroxide value; AnV—anisidine value; TOTOX—total oxidation index; AV—acid value), areas of oil droplets (ODs) and color parameters (L*—lightness; a*—redness; b*—yellowness) of aquafaba-based mayonnaise samples with blends of refined rapeseed oil and cold-pressed rapeseed oil (MRO), cold-pressed sunflower oil (MSO), cold-pressed linseed oil (MLO), and cold-pressed camelina oil (MCO); MT1—commercial egg yolk mayonnaise from producer 1; MT2—commercial egg yolk mayonnaise from producer 2; MV—commercial vegan mayonnaise.

The entire data set was visualized by a bi-plot (combined scores and loadings plot for two components) and presented in Figure 4.

As can be seen, the QUENCHER-DPPH (0.8486), QUENCHER-ABTS (0.8497), AV (0.0010), L* (0.8625), and a* (0.0146) of mayonnaise samples had positive loadings on the PC1, whereas the PV (−0.9169), AnV (−0.9388), TOTOX indexes (−0.9656), ODs size (−0.9123), and b* (−0.3271) were the variables with negative loadings on PC1. However, all variables (except AnV) revealed loadings on the negative dimension of PC2 (−0.0045–−0.9819). It can be noted that QUENCHER-DPPH, QUENCHER-ABTS, AV, L* and a* were the variables with positive loadings on PC1 and negative loadings on PC2. On the contrary, AnV revealed loadings on the negative dimension of PC1 and positive on PC2. However, b*, PV, TOTOX and ODs were the features with negative loadings on PC1 and PC2.

The PCA graph depicted that the two vegan mayonnaises (the commercial MV and MLO sample made from a blend of RRO and CPLO) with the lowest RSA (QUENCHER-DPPH = 589–626 μmol TE/100 g, QUENCHER-ABTS = 1371–1674 μmol TE/100 g) and L* (41.4–41.5) values and the highest oxidative parameters (PV = 2.27–4.61 mEq O_2_/kg, AnV = 2.95–3.07, TOTOX = 7.49–12.29) and area of ODs (56.76–101.40 μm^2^) were located to the left in the score bi-plot and had negative values for PC1. Three prepared vegan emulsions (MSO, MCO and MRO) and two commercial egg yolk mayonnaises (MT1 and MT2) with high radical scavenging properties (QUENCHER-DPPH = 643–828 μmol TE/100 g, QUENCHER-ABTS = 1836–3320 μmol TE/100 g) and more lightness (L* = 44.5–48.7) were situated at the right in the score bi-plot and had positive values for PC1. Consequently, the studied samples could be divided into three groups based on their distribution on the PCA graph. The yellowest commercial plant-based mayonnaise (MV) with the longest distance from other samples had the highest of all the oxidative parameters (PV, AnV, TOTOX, AV), ODs area and the lowest radical scavenging properties determined by two analytical methods (QUENCHER-DPPH and QUENCHER-ABTS). Two traditional mayonnaises containing egg yolk (MT1 and MT2) characterized by the highest RSA (QUENCHER-DPPH = 692–828 μmol TE/100 g, QUENCHER-ABTS = 2808–3320 μmol TE/100 g), the lowest ODs (4.44–4.47 μm^2^) and with the same AV results (0.49 mg KOH/g) created a distinct cluster. Additionally, four egg-free mayonnaises made from blends of RRO and cold-pressed vegetable oils with moderate radical scavenging properties (QUENCHER-DPPH = 626–690 μmol TE/100 g, QUENCHER-ABTS = 1674–2121 μmol TE/100 g), oxidative status (PV = 1.19–2.27 mEq O_2_/kg, AnV = 1.12–2.95, TOTOX = 3.93–7.49), and ODs areas (14.29–56.75 μm^2^) as well as the lowest AV (0.11–0.26 mg KOH/g) and a* (−0.3–0.9) values were separated from the other studied samples and located at the upper the A1 axis.

It is noteworthy that there was a high correlation between the QUENCHER-DPPH and QUENCHER-ABTS results for all studied mayonnaises (r = 0.9427). This suggests that antioxidants present in the studied samples have the ability to scavenge both stable DPPH radical and ABTS cation radical.

Moreover, RSA values of the discussed mayonnaises were positively correlated with their AV data (r = 0.3177 and 0.4319) as well as color parameters, such as lightness (L*, r = 0.6238 and 0.6894) and redness (a*, r = 0.2615 and 0.4544), but negatively associated with oxidative parameters (PV, AnV and TOTOX, r = −0.6453–−0.7549) and ODs sizes (r = −0.6432–−0.7315). These high negative correlations between oxidative parameters and radical scavenging properties of the studied emulsions indicated the significant contribution of the antioxidant potential of samples to enhance their oxidation status. The lower PV values can be explained by the hindered oxidation of the lipid fraction in the mayonnaise samples, and thus the delay in their deterioration, due to the presence antioxidants and higher RSA values.

As expected, the PV results for seven mayonnaises contributed significantly and positively with the AnV (0.7800) and TOTOX indexes (0.9843). Additionally, a high correlation coefficient (r = 0.8781) was found between AnV and TOTOX data for these samples.

Interestingly, there were high positive correlations between the amounts of primary and secondary oxidation products in the analyzed samples and ODs (r = 0.8162–0.9117). These positive correlations indicate that emulsions with smaller areas of ODs had a better oxidative stability. Nevertheless, the area of ODs for mayonnaises was negatively associated with their lightness (L*, r = −0.7916). However, negative correlation coefficients (r = −0.7470–−0.8583) for the relationships between PV, AnV, TOTOX and L* were observed.

In contrast, there were positive correlations between the free fatty acids content and color parameters of the investigated samples (AV–a*, r = 0.8540 and a*–b*, r = 0.7187).

The results obtained by PCA indicated the differences in radical scavenging and physicochemical properties of two yolk egg mayonnaises, one commercial vegan mayonnaise, and four aquafaba-based samples made from blends of RRO and cold-pressed vegetable oils. This grouping of the samples suggests that the used ingredients in mayonnaise samples and technological conditions were responsible for their quality.

## 4. Conclusions

Aquafaba-based vegan mayonnaises with blends of RRO and different cold-pressed oils were made. This research paper has presented that aquafaba from chickpeas is a suitable alternative emulsifier with high radical scavenging properties for the production of egg-free vegan emulsions. Moreover, the other ingredients of the proposed mayonnaise formulations, mainly nutritional yeast and mustard, had high radical scavenging characteristics. Additionally, cold-pressed vegetable oils used as functional ingredients in developing vegan egg-free mayonnaises were rich sources of unsaturated fatty acids and natural bioactive compounds with scavenging radical properties.

The new aquafaba-based emulsions containing blends of RRO with different cold-pressed oils had higher radical scavenging properties and oxidative stability, similar color parameters, and a smaller size of ODs than commercial vegan mayonnaise (MV). A significant contribution of the radical scavenging properties of the prepared vegan mayonnaises to enhance their oxidation status was observed. Additionally, emulsions with smaller areas of ODs had better oxidative stability.

The results of PCA indicated that the radical scavenging and physicochemical properties of aquafaba-based emulsions, commercial vegan mayonnaise (MV) and traditional emulsions containing egg yolk (MT1 and MT2) as the main emulsifier differ significantly.

Novel plant-based emulsions could be a promising alternative to egg-based mayonnaises and salad dressing. Further investigations should be performed to determine the effect of aquafaba on the textural and sensory properties and consumer acceptability of mayonnaise.

## Figures and Tables

**Table 1:** Ingredients of different formulations of vegan mayonnaise samples.

Ingredient	MRO (g/100 g)	MSO (g/100 g)	MLO (g/100 g)	MCO (g/100 g)
Aquafaba	23.70	23.70	23.70	23.70
Mustard	1.25	1.25	1.25	1.25
Nutritional yeast	0.10	0.10	0.10	0.10
Vinegar	0.65	0.65	0.65	0.65
Sugar	0.15	0.15	0.15	0.15
Salt	0.15	0.15	0.15	0.15
RRO	67.00	67.00	67.00	67.00
CPRO	7.00	–	–	–
CPSO	–	7.00	–	–
CPLO	–	–	7.00	–
CPCO	–	–	–	7.00

MRO—mayonnaise with a blend of refined rapeseed oil (RRO) and cold-pressed rapeseed oil (CPRO); MSO—mayonnaise with a blend of refined rapeseed oil (RRO) and cold-pressed sunflower oil (CPSO); MLO—mayonnaise with a blend of refined rapeseed oil (RRO) and cold-pressed linseed oil (CPLO); MCO—mayonnaise with a blend of refined rapeseed oil (RRO) and cold-pressed camelina oil (CPCO).

**Table 2:** Emulsifying activity index (EAI), emulsion stability index (ESI), and protein content in aquafaba and egg yolk.

Emulsifier Type	EAI ± SD (m^2^/g)	ESI ± SD (min)	Protein ± SD (%)
Aquafaba	13.75 ± 0.69 ^b^	20.92 ± 0.61 ^a^	1.26 ± 0.05 ^a^
Egg yolk	1.78 ± 0.07 ^a^	2385 ± 103 ^b^	16.12 ± 0.47 ^b^

*n* = 3; SD—standard deviation; Different letters (a, b) within the same column indicate significant differences between emulsifying parameters (EAI—emulsifying activity index, ESI—emulsifying stability index) and protein content in emulsifiers (Tukey’s post hoc test, *p* < 0.05).

**Table 3:** Fatty acid compositions of oils used in making vegan mayonnaises.

Oil Type	C16:0 ± SD (%)	C18:0 ± SD (%)	C18:1 ± SD (%)	C18:2 ± SD (%)	C18:3 ± SD (%)	C20:0 ± SD (%)	C20:1 ± SD (%)	ΣSAFA (%)	ΣMUFA (%)	ΣPUFA (%)
RRO	4.4 ± 0.1 ^a^	1.8 ± 0.1 ^a^	62.2 ± 0.2 ^d^	19.3 ± 0.1 ^b,c^	9.2 ± 0.1 ^c^	0.7 ± 0.0 ^c^	1.4 ± 0.0 ^c^	6.9	63.6	28.5
CPRO	4.8 ± 0.0 ^b^	1.6 ± 0.0 ^a^	63.4 ± 0.1 ^e^	19.4 ± 0.0 ^c^	8.0 ± 0.1 ^b^	0.6 ± 0.0 ^c^	1.2 ± 0.0 ^b^	7.0	64.6	27.4
CPSO	6.6 ± 0.1 ^e^	3.7 ± 0.1^c^	29.0 ± 0.0 ^c^	58.9 ± 0.1 ^d^	0.1 ± 0.0 ^a^	0.3 ± 0.0 ^b^	0.1 ± 0.0 ^a^	10.6	29.1	59.0
CPLO	5.3 ± 0.0 ^c^	2.5 ± 0.0 ^b^	18.8 ± 0.1 ^b^	19.2 ± 0.0 ^b^	30.5 ± 0.1 ^d^	1.7 ± 0.1 ^d^	15.0 ± 0.1 ^d^	9.5	33.8	49.7
CPCO	5.5 ± 0.0 ^d^	4.1 ± 0.1 ^d^	15.6 ± 0.0 ^a^	16.3 ± 0.0 ^a^	57.5 ± 0.1 ^e^	0.1 ± 0.0 ^a^	0.1 ± 0.0 ^a^	9.7	15.7	73.8

*n* = 5; SD—standard deviation; Different letters (a–e) within the same column indicate significant differences between the percentages of fatty acids of vegetable oils: RRO—refined rapeseed oil, CPRO—cold-pressed rapeseed oil, CPSO—cold-pressed sunflower oil, CPLO—cold-pressed linseed oil, CPCO—cold-pressed camelina oil (Tukey’s post hoc test, *p* < 0.05); SAFA—saturated fatty acids; MUFA—monounsaturated fatty acids; PUFA—polyunsaturated fatty acid.

**Table 4:** Radical scavenging activity of mayonnaise ingredients determined by DPPH and ABTS assays.

Ingredient	DPPH ± SD (μmol TE/100 g)	ABTS ± SD (μmol TE/100 g)
Aquafaba	437 ± 22 ^b^	2097 ± 22 ^b^
Nutritional yeast	1687 ± 60 ^d^	9192 ± 238 ^d^
Mustard	1129 ± 16 ^c^	4985 ± 107 ^c^
RRO	387 ± 8.3 ^b^	669 ± 29 ^a^
CPRO	391 ± 19 ^b^	738 ± 25 ^a^
CPSO	290 ± 13 ^a^	588 ± 15 ^a^
CPLO	272 ± 4.5 ^a^	602 ± 27 ^a^
CPCO	387 ± 4.5 ^b^	706 ± 34 ^a^

*n* = 5; SD—Standard Deviation; Different letters (a–d) within the same column indicate significant differences between radical scavenging activity of samples: RRO—refined rapeseed oil, CPRO—cold-pressed rapeseed oil, CPSO—cold-pressed sunflower oil, CPLO—cold-pressed linseed oil, CPCO—cold-pressed camelina oil (Tukey’s post hoc test, *p* < 0.05).

**Table 5:** Oxidation parameters of mayonnaises.

Mayonnaise Type	PV ± SD (mEq O_2_/kg)	AnV ± SD	TOTOX Index	AV ± SD (mg KOH/g)
MRO	1.43 ± 0.06 ^c^	1.39 ± 0.08 ^c^	4.25	0.14 ± 0.01 ^a,b^
MSO	1.90 ± 0.06 ^d^	1.12 ± 0.06 ^b^	4.92	0.26 ± 0.01 ^c^
MLO	2.27 ± 0.10 ^f^	2.95 ± 0.06 ^d^	7.49	0.11 ± 0.01 ^a^
MCO	1.19 ± 0.01 ^b^	1.55 ± 0.09 ^c^	3.93	0.17 ± 0.01 ^b^
MT1	2.09 ± 0.07 ^e^	1.19 ± 0.08 ^b,c^	5.37	0.49 ± 0.01 ^d^
MT2	0.82 ± 0.01 ^a^	0.76 ± 0.05 ^a^	2.40	0.49 ± 0.02 ^d^
MV	4.61 ± 0.08 ^g^	3.07 ± 0.12 ^d^	12.29	0.52 ± 0.03 ^d^

*n* = 3; SD—Standard Deviation; Different letters (a–g) within the same column indicate significant differences between oxidation parameters (PV—peroxide value; AnV—anisidine value; TOTOX—total oxidation index; AV—acid value) of aquafaba-based mayonnaises with blends of refined rapeseed oil and cold-pressed rapeseed oil (MRO), cold-pressed sunflower oil (MSO), cold-pressed linseed oil (MLO), and cold-pressed camelina oil (MCO); MT1—commercial egg yolk mayonnaise from producer 1; MT2—commercial egg yolk mayonnaise from producer 2; MV—commercial vegan mayonnaise (Tukey’s post hoc test, *p* < 0.05).

**Table 6:** Color of mayonnaises.

Mayonnaise Type	L* ± SD	a* ± SD	b* ± SD
MRO	48.7 ± 0.2 ^e^	0.9 ± 0.2 ^c^	10.9 ± 0.4 ^c^
MSO	44.5 ± 0.1 ^b^	−0.3 ± 0.1 ^a^	7.1 ± 0.3 ^a^
MLO	41.5 ± 0.3 ^a^	0.2 ± 0.1 ^b^	9.2 ± 0.2 ^b^
MCO	47.1 ± 0.5 ^c^	0.5 ± 0.1 ^b^	6.7 ± 0.1 ^a^
MT1	47.9 ± 0.1 ^d^	3.4 ± 0.1 ^f^	11.2 ± 0.1 ^c^
MT2	47.7 ± 0.2 ^d^	2.2 ± 0.2 ^d^	9.0 ± 0.4 ^b^
MV	41.4 ± 0.2 ^a^	2.6 ± 0.2 ^e^	11.3 ± 0.4 ^c^

*n* = 5; SD—Standard Deviation; Different letters (a–f) within the same column indicate significant differences between color parameters (L*—lightness; a*—redness; b*—yellowness) of aquafaba-based mayonnaise samples with blends of refined rapeseed oil and cold-pressed rapeseed oil (MRO), cold-pressed sunflower oil (MSO), cold-pressed linseed oil (MLO), and cold-pressed camelina oil (MCO); MT1—commercial egg yolk mayonnaise from producer 1; MT2—commercial egg yolk mayonnaise from producer 2; MV—commercial vegan mayonnaise (Tukey’s post hoc test, *p* < 0.05).

## Data Availability

The data presented in this study are available on request from the corresponding author. The data are not publicly available due to privacy or ethical restrictions.
